# Auxin Orchestrates Germ Cell Specification in *Arabidopsis*

**DOI:** 10.3390/ijms26073257

**Published:** 2025-04-01

**Authors:** Tian-Ying Yu, Ping Wang, Yue Lv, Bo Wang, Ming-Ri Zhao, Xin-Wei Dong

**Affiliations:** College of Life Sciences, Yantai University, Yantai 264005, China

**Keywords:** Auxin, Sporocyteless/Nozzle (SPL/NZZ), archesporial cells, megaspore mother cells (megasporocyte), microspore mother cells, germ cell specification

## Abstract

The initiation and specification of germline cells are crucial for plant reproduction and the continuity of species. In *Arabidopsis thaliana*, auxin plays a vital role in guiding the transition of somatic cells into germline fate, orchestrating the specification of both male archesporial cells and female megaspore mother cells. This process is regulated through interaction with the transcription factor Sporocyteless/Nozzle, which forms a feedback mechanism that modulates germ cell specialization. Auxin biosynthesis, polar transport, and signal transduction pathways collectively ensure the accurate determination of germ cell fate. Furthermore, the coordination of auxin signaling with epigenetic regulation and miRNA-mediated control fine-tunes the differentiation between germline and somatic cells. This review discusses the mechanisms underlying auxin-guided germ cell specification. It proposes future research directions, including studies on PIN-FORMED-mediated polar transport, the role of the YUCCA family in auxin biosynthesis, and the involvement of the Transport Inhibitors Response 1/Auxn Signaling F-Box-Auxin Response Factor (TIR1/AFB-ARF) signaling pathway in germ cell fate determination. These insights will enhance our understanding of plant reproductive biology and provide new strategies for crop breeding.

## 1. Introduction

Germ cell initiation and specification is pivotal in the reproductive development of multicellular organisms, bridging the sporophytic and gametophytic generations [1]. Germ cells, often known as megaspore and microspore mother cells, undergo meiosis to produce haploid gametes and are vital for genetic continuity [2]. In angiosperms, male archesporial cells differentiate into microspore mother cells, while female sporogenous cells develop into megaspore mother cells. These cells then proceed to meiosis, resulting in the formation of microspores and megaspores [3,4,5,6].

In contrast to animals, where primordial germ cells are determined early during embryogenesis [7,8], the specification of germ cells in higher plants occurs much later, arising from somatic cells after the development of the embryo [9,10]. This process is tightly regulated by a complex genetic network [11]. Recent studies have highlighted auxin as a key regulator that orchestrates the transition of somatic cells to germline fate [12,13].

Auxin plays indispensable roles throughout plant developmental processes, including organ formation and cell differentiation [14]. In reproductive development, auxin influences floral organ formation and seed development [15,16] and plays a significant role in both sporophytes and gametophytes development [17,18,19]. Recent research has advanced our understanding of auxin’s crucial role in germline specification, especially its influence on the transition from somatic cells into germ cells [12,13,20].

This review explores the significant roles of auxin in regulating germ cell specification, focusing on both male (archesporial cell) and female germ cells. We investigate the shared pathways that govern these processes, as well as the potential molecular mechanisms and feedback loops involved. By synthesizing recent findings, we aim to establish a new theoretical framework for understanding auxin’s role in germline specification and suggest experimental avenues for further investigation.

## 2. Auxin Homeostasis: Biosynthesis, Polar Transport, Signal Transduction, Metabolism, and Conjugation

Auxin homeostasis is maintained through a complex network involving its biosynthesis, polar transport, metabolism, and conjugation. Auxin is primarily synthesized via multiple pathways, notably the Tryptophan Aminotransferase of *Arabidopsis* (TAA1)/Tryptophan Aminotransferase Related 2 (TAR2)-YUCCA (YUC) pathway [21,22,23,24], which is subject to precise regulation by hormonal and environmental cues. Its polar transport, chiefly mediated by PIN-FORMED (PIN) efflux carriers [25,26], establishes dynamic distributions that are essential for processes such as germ cell specification. In parallel, auxin levels are finely tuned by metabolic enzymes and conjugating proteins, such as those in the GH3 family [27,28], that modulate auxin activity by promoting its oxidation or conjugation, thereby serving as critical control points during development and stress adaptation. Moreover, auxin signaling extends beyond the canonical TIR1/AFB (Transport Inhibitor Response 1/Auxin-Signaling F-Box)-mediated pathway [29,30]; recent studies have revealed the pivotal role of the cell surface ABP1-TMK (Auxin-binding protein 1-transmembrane kinase 1) complex in activating ROP (RHO of plant) GTPase signaling cascades [31,32], along with additional non-canonical signaling mechanisms. Collectively, these regulatory layers integrate to ensure precise auxin distribution and responsiveness, highlighting its fundamental role in orchestrating the intricate developmental processes of flowering plants.

### 2.1. Auxin Biosynthesis: Key Genes and Pathways

The biosynthesis of auxin constitutes a fundamental aspect of auxin activity regulation within plant tissues [33]. The predominant auxin in plants, indole-3-acetic acid (IAA), is synthesized through two principal pathways [33,34,35]: the tryptophan-dependent pathway [14] and the tryptophan-independent pathway [36]. The tryptophan-dependent pathways have been the subject of more in-depth research, involving key enzymes such as TAA1 [21] and members of the YUC family [22,23]. Notably, auxin biosynthesis responds dynamically to developmental cues and environmental conditions [37,38]. Feedback mechanisms are required for regulating biosynthetic and transport genes [24,29].

### 2.2. Auxin Transport: Polar Distribution and Accumulation

The polar transport mechanisms are indispensable for establishing auxin maxima within plant tissues [39]. Key players in this process are PIN proteins, which function as efflux carriers to direct auxin flow between cells [25,26,40,41]. Multiple studies have demonstrated that PIN proteins exhibit a polarized distribution along the plasma membrane, which is crucial for guiding auxin transport [40,42,43]. Moreover, the serine/threonine kinase PINOID (PID) phosphorylates PIN proteins, thereby modulating their polar localization and ensuring effective auxin transport [25,43,44]. The interplay between PIN-mediated efflux and AUXIN1/LIKE-AUX1 (AUX/LAX)-mediated influx facilitates the ongoing redistribution of auxin, thereby promoting local accumulation and the establishment of auxin maxima [45,46].

### 2.3. Auxin Signal Transduction: From Perception to Transcription

The fundamental elements of the auxin signaling pathway consist of the TIR1/AFB receptors family [14,47], which functions as the components of an E3 ubiquitin ligase complex responsible for the degradation of Aux/IAA proteins [48]. In the presence of auxin, the TIR1/AFB receptors associate with auxin, triggering the degradation of Aux/IAA proteins and releasing ARFs to initiate the transcription of auxin-responsive genes [49,50,51,52]. The interplay between ARFs and Aux/IAA proteins determines the specificity of auxin signaling, ensuring the correct activation or inhibition of target genes in response to auxin levels [53]. ABP1 can sense and bind auxin. Together with the receptor-like protein kinase TMK, ABP1 forms a cell surface auxin-sensing complex that activates the Rho family of small GTPases (ROPs) signaling pathway. This complex plays a critical role in plant development and morphogenesis by regulating non-transcriptional cytoplasmic responses and associated fundamental physiological processes [31,32].

### 2.4. Dynamic Equilibrium of Auxin: Integration of Biosynthesis, Transport, Signal Transduction, Metabolism, and Conjugation

The processes of auxin biosynthesis, transport, signaling, metabolism, and conjugation are interconnected, maintaining a dynamic equilibrium that ensures precise spatial and temporal accumulation of auxin [17]. Feedback regulation and integration of these processes are essential for coordinating developmental programs, with key proteins such as TAA1, YUCs, PINs, TIR1/AFB, ABP1-TMK, and ARFs orchestrating the balance [31,32,54].

## 3. Auxin Orchestrates the Specification of Germ Cells in the Anther

**Figure 1:**
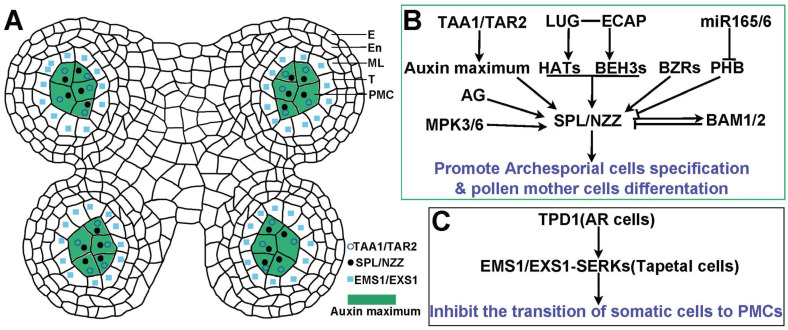
Male germ cell specification in *Arabidopsis*. (**A**) A schematic cross-section of an anther lobe illustrating tissue layers and the positioning of pollen mother cells (PMCs). The tissue layers include the epidermis (E), endothecium (En), middle layer (ML), and tapetum (T), with PMCs located at the center of the locule. Auxin maxima highlighted in green. (**B**) Auxin maxima, established through TAA1/TAR1 activity, serve as environmental signals. The *Arabidopsis* MADS-box C-class protein AG directly interacts with a CArG-box-like motif in the 3′ region of SPL/NZZ to activate its expression. BZR1/BES1 directly binds to the G-box of the SPL/NZZ promoter to induce SPL/NZZ expression. Additionally, LUG-ECAP forms a transcriptional activator complex that recruits HATs and the BZR1 family member BEH3, further enhancing SPL/NZZ expression. Moreover, BAM1/2 restricts the specific expression of SPL/NZZ in microsporocytes, while SPL/NZZ expression, in turn, promotes the preferential expression of BAM1/2 in the microspore mother cell. Furthermore, MPK3/6 phosphorylates SPL/NZZ to enhance its stability. Collectively, these factors orchestrate SPL/NZZ activity to direct the specification of archesporial cells and promote the differentiation of PMCs. (**C**) TPD1 is secreted from archesporial cells (AR cells) and recognized by the EMS1/EXS1-SERKs receptor complex in tapetal cells; this signal pathway inhibits the transition of somatic cells into PMCs (pollen mother cells), thereby maintaining proper cell identity within the anther. Abbreviations: TAA1, Tryptophan Aminotransferase of *Arabidopsis* 1; TAR2, Tryptophan Aminotransferase-Related 2; LUG, Gro/Tup1 family corepressor LEUNIG; ECAP, EAR motif-containing adaptor protein; HATs, histone acetyltransferases; miR165/6, microRNA 165/microRNA166; PHB, PHABULOSA; BZR1, Brassinazole resistant transcription factor 1; BEH3, BES1/BZR1 Himolog3; AG, C-class gene AGAMOUS in ABC-BOX model; MPK3/6, Protein kinases MPK3 and MPK6; SPL/NZZ, Sporocyteless/Nozzle; BAM1/2, Barely Any Meristem (BAM) 1 and 2; TPD1, Tapetum Determinant 1; EMS1/EXS1, Excess Microsporocytes 1/Extra Sporogenous cell 1; SERKs, Somatic Embryogenesis Receptor-Like Kinases.

The specification of male germ cells is a crucial event in the developmental programs of the anther. In *Arabidopsis thaliana*, the anther development progression is intricate and categorized into fourteen stages based on histological characteristics [55]. This process begins with generating three-layered stamen primordia, comprised of somatic cells. Stages one through five represent the pre-meiotic phase, during which four radially symmetrical microsporangia are formed (Figure 1A), marking the culmination of germ cell specification [56]. Male germ cells arise from somatic cells in the L2 layer at each corner of the anther primordium, where they undergo division and specification into inner archesporial cells, commonly referred to as germ cells, surrounded by somatic layers, including the endothecium, middle layer, and tapetum [56]. This specification marks the initiation of microspore mother cells, which will enter meiosis to produce haploid pollen grains [56]. The transition from somatic to germline cell identity is orchestrated by genetic determinants, particularly SPL/NZZ, and local environmental cues with auxin design [3,57,58]. The involvement of SPL/NZZ in germline specification during early developmental stages has been well-documented, highlighting the connection between genetic regulation and auxin signaling.

### 3.1. Auxin Biosynthesis and Specification of Male Germ Cells

During the anther development, auxin dynamically accumulates in germline cell types, including archesporial cells, primary germ cells, and pollen mother cells [59]. Initially, auxin is uniformly distributed throughout the anther primordium. By the third stage, auxin concentration increases at the center, though still at low levels. In stage five, a notable peak in auxin concentration is observed in the pollen mother cells, resulting in significant accumulation in each lobe in the anther (Figure 1A) [12]. This auxin maxima in germline cells underscores its critical role in regulating cell fate within the anther, establishing auxin as a key player in germ cell specification [60]. The formation of local auxin maxima provides positional cues, distinguishing germline progenitor cells from adjacent somatic cells [38,61]. Auxin directs the specification of germ cells while promoting the differentiation of surrounding somatic tissues, such as the tapetum and middle layer [12].

Auxin biosynthesis in the anther is intricately regulated to maintain appropriate concentrations for directing cellular differentiation at distinct developmental stages [14]. In *Arabidopsis,* TAA1 and TAR2 are essential for the conversion of L-tryptophan into indole-3-pyruvic acid, a key intermediary in the biosynthesis of IAA [24,62]. The expression of *TAA1* and *TAR2* follows tissue-specific patterns, and their activity during the anther development is crucial for establishing local auxin concentrations that guide germ cell specification [22].

Initially, *TAA1* and *TAR2* are expressed in the inflorescence meristem [12,24]. As the anther primordium undergoes differentiation, their expression becomes increasingly localized to germline cells, particularly microspore mother cells at the fifth stage [12]. In the *taa1 tar2-2* mutant, microspore mother cells exhibit morphological defects, and somatic layers show disorganization, indicating that mutations in *TAA1/TAR2* disrupt both germ cell initiation and microspore mother cell formation. The expression profiles of *TAA1* and *TAR2*, along with auxin accumulation, are closely linked to the proper specification of male germ cells [12].

### 3.2. Auxin-Mediated Regulation of SPL/NZZ in Male Germ Cells Specification

Precise temporal and spatial regulation of auxin is essential for orchestrating germ cell specification. In early anther development, auxin levels remain low, promoting somatic cell proliferation and anther lobe formation [12]. As development proceeds, auxin is redistributed toward specific tissues, initiating germ cell specification. The temporal expression of *TAA1/TAR2* is critical for ensuring auxin accumulation in the right areas at the appropriate time, guiding the specification of archesporial cells [12]. The precise control of auxin maximum and SPL/NZZ activity ensures the correct timing of germ cell differentiation, avoiding premature or delayed differentiation [12,63,64,65].

#### 3.2.1. Transcriptional Regulation and Feedback Mechanisms Between Auxin and SPL/NZZ

Crosstalk between SPL/NZZ and auxin signaling reveals a complex interplay crucial for germ cell specification. The expression of *SPL/NZZ* is regulated by auxin-related signaling pathways [66]. In the *taa1 tar2-2* double mutant, *SPL/NZZ* promoter activity is significantly downregulated, suggesting a direct role for TAA1/TAR2 in local auxin biosynthesis and the activation of *SPL/NZZ* expression [12,67]. The ectopic expression of *SPL/NZZ* in germ cells partially rescues the abortion phenotype in these mutants [68], indicating the importance of SPL/NZZ for initiating archesporial cells [67]. Overaccumulation of *TAA1/TAR2* transcripts in *spl* mutants implies a feedback mechanism where SPL/NZZ represses the expression of *TAA1/TAR2*, demonstrating a dynamic feedback loop that ensures proper regulation of both SPL/NZZ and auxin biosynthesis [69].

#### 3.2.2. Transcriptional Regulation of SPL/NZZ

The transcription factor SPL/NZZ operates as a key regulator in the specification of germ cells and the initiation of sporogenesis in *Arabidopsis* (Figure 1B) [3,11,12,70]. *SPL/NZZ* expression is regulated by the SET Domain Group 2 (SDG2), a lysine methyltransferase that trimethylates histone H3 at H3K4, enhancing the expression of the *SPL/NZZ* [71]. Several transcription factors, such as the *Arabidopsis* MADS-box C-class protein AGAMOUS (AG) [72] and members of the Brassinazole Resistant 1 (BZR1)/bri1 EMS Suppressor 1 (BES1) family [73,74], also promote *SPL/NZZ* expression. The interaction between BES1/BZR1 HOMOLOG 3 (BEH3), a member of the BZR1 family, and the *SPL/NZZ* promoter recruits the transcriptional activator complex, EAR-motif-containing adaptor proteins (ECAP), and the corepressor LEUNIG (LUG), which activate *SPL/NZZ* expression [75]. AG interacts directly with a CArG-box-like motif localized in the 3′ region of *SPL/NZZ.* It is not required for maintaining *SPL/NZZ* expression [72]. This transcriptional regulation, coupled with microRNA165/6 (miRNA165/6) and the feedback loop involving Barely Any Meristem 1/2 (BAM1/2) [76], ensures the correct timing and localization of *SPL/NZZ* expression in microspore mother cells [75]. Additionally, research by Hord et al. (2008) highlights that MAPK3/6 phosphorylates SPL/NZZ, enhancing the stability and activity of SPL/NZZ [77].

### 3.3. Synergistic Regulations Between Auxin and TPD1-EMS1 Signaling Pathways

Auxin interacts with other signaling pathways, such as cytokinin, gibberellin, and abscisic acid, to regulate germ cell specification. Auxin works synergistically with the TPD1-EMS1 (Tapetum Determinant 1-Excess Microsporocytes 1) pathway to balance the differentiation of germ and somatic cells [78]. In ems1 or tpd1 mutants, tapetal cells are absent, leading to an over-proliferation of germ cells and male sterility. The TPD1-EMS1 signaling pathway prevents tapetal cells from differentiating into germline cells, facilitating communication between archesporial cells and their surrounding somatic cells [79]. It is proposed that auxin modulates the expression of EMS1 and TPD1 by interacting with various components of this signaling pathway, maintaining the equilibrium between germ and somatic cells, and ensuring the proper formation of archesporial cells (Figure 1C) [78,79]. This underscores the important role of auxin in fine-tuning cellular composition during the anther development.

## 4. Auxin Determines Female Germline Cell Specification

In flowering plants, the female germline arises from the specification of somatic cells within the ovule [11]. During the early stages of ovule development, a specific subepidermal cell is selected through intricate molecular mechanisms to transition into one megaspore mother cell (MMC) [80,81]. This megaspore mother cell rapidly becomes morphologically distinct from surrounding sporophytic cells. It then undergoes meiosis to produce a tetrad of four haploid megaspores [5,57,82,83,84]. In most angiosperms, three of these megaspores degenerate, leaving a single functional megaspore at the base of the tetrad. This functional megaspore subsequently undergoes three rounds of nuclear mitosis, followed by nucleus migration and cellularization. This process ultimately results in the formation of a seven-celled, eight-nucleate female gametophyte [5,82,85], which is essential for successful fertilization and seed development [86]. The transition from somatic to germline cells is regulated by genetic and hormonal signals [14]. A key transcription factor, SPL/NZZ, governs the specification of female germline cells [3]. It is expressed in the L1 epidermal layer of the nucellus in a pattern that aligns with auxin maxima, implying its interaction with auxin in the specification of megaspore mother cell [13,86]. The SPL/NZZ-controlled signaling pathway operates non-cell-autonomously to regulate female germline specification [13].

### 4.1. Dynamic Distribution of Auxin and Specification of Megaspore Mother Cell

The formation of the megaspore mother cell in the ovule represents a critical step in the development of female gametophytes. This process relies on a localized auxin peak, which serves as a spatial cue for germline cells specification [13]. Auxin accumulates in the epidermal cells of the nucellus through tightly controlled polar transport mechanisms, creating concentration maxima that identify the subepidermal cell destined to become a megaspore mother cell. This auxin-driven process is essential for the precise selection and formation of a single megaspore mother cell [13], is the prerequisite for gametophyte formation and fertility.

**Figure 2:**
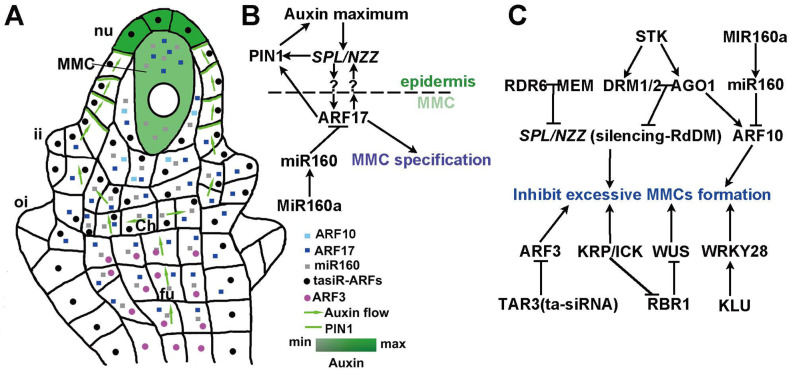
Specification of female germline cell in *Arabidopsis*. (**A**) Schematic representation of an *Arabidopsis* ovule primordium illustrating auxin distribution and key regulatory proteins essential for the formation of megaspore mother cell (MMC). The green shading indicates auxin distribution, with deep green denoting auxin maxima located in the apex epidermal cells of the nucellus (nu), and light green highlighting the MMC situated in subepidermal cells of the nucellus, alongside surrounding epidermal cells that play a role in its regulation. In the ovule, megaspore mother cell development is initiated. Auxin, mediated by PIN1, moves from the funiculus (fu) and chalazal end (Ch) along the epidermis of the nucellus (nu) to the apex and the subepidermal MMC, reaching its maximum in the epidermal cells at the apex of the nucellus. Meanwhile, the inner integument (ii) and outer integument (oi) begin to develop. (**B**) Auxin maxima facilitate MMC specification by modulating the transcription factor SPL/NZZ. This process is enhanced by PIN1-mediated auxin transport and regulated by ARF17, while miR160 and MIR160a control ARF17 expression to ensure the formation of a single MMC. (**C**) STK represses SPL/NZZ transcription via its downstream components AGO1 and DRM1/2 through RNA-dependent DNA methylation (RdDM) mechanisms, while the RDR6-MEM pathway epigenetically inhibits *SPL/NZZ* expression. A trans-acting siRNA pathway (TAR3) confines *ARF3* expression to a specific region of the nucellus. Concurrently, RBR1 suppresses *WUS* expression to inhibit the production of additional MMCs, while KRP/ICK proteins restrict cell division, further preventing excessive MMC formation. In addition, KLU activates the WRKY28 transcription factor, which also limits the development of supernumerary MMCs. Moreover, ARF10, expressed in cells adjacent to the MMC, is tightly regulated by miR160 to ensure that these surrounding cells do not adopt an MMC identity. Collectively, these regulatory mechanisms work synergistically to guarantee that only one MMC is generated per ovule. Abbreviations: PIN1, PIN-FORMED; SPL/NZZ, Sporocyteless/Nozzle; ARF17, Auxin Response Factor 17; miR160, microRNA 160; STK, SEEDSTICK; RDR6, RNA-Dependent RNA Polymerase 6; NEM, MNEME; AGO1, ARGONAUTE1; DRM1/2, Domains Rearranged Methyltransferases 1/2; ARF10, Auxin Response Factor 10; KRP/ICK, KIP-Related Protein and Interactors/Inhibitors of Cyclin-Dependent Kinase; ARF3, Auxin Response Factor 10; WUS, WUSCHEL; WRKY28, containing WRKYGOK motif transcript factor 28; TAR3 (ta-siRNA), trans-acting siRNA pathway restricts ARF3 expression; RBR1, Retinoblastoma-Related1; KLU, KLUH/CYP78A5.

The specification of the female germline cell depends on the formation of a localized auxin maximum and is achieved by the polar transport of auxin via PIN proteins (Figure 2A) [87]. Specifically, PIN1, expressed in the sporophytic tissues [88]. Polar localization of PIN proteins in the basal and apical epidermal layers ensures the directional transport of auxin [39,88], leading to the polarized accumulation of auxin at the apex of the nucellus and designing the megaspore mother cell beneath it (Figure 2) [13,88]. Experiments using *pin1-5* mutants and wild-type plants treated with the auxin transport inhibitor N-1-naphthylphthalamic acid (NPA) have shown that disrupting this polar transport results in the formation of multiple MMC-like cells, underscoring the importance of a precise auxin maximum for ensuring a unique MMC [13].

### 4.2. Auxin Signaling Pathways in Female Germline Specification

#### 4.2.1. ARFs Function in Female Germline Specification

Auxin orchestrates female germline cell specification through a well-defined signaling pathway, where ARF10 and ARF17 are key components that convert auxin signals to initiate this process [13].

*ARF17* transcripts are detected in the megaspore mother cell, chalaza, and funiculus of wild-type ovules; however, in *pARF17::ARF17-GFP* transgenic plants, GFP signals are absent from these tissues. Immunofluorescence analysis has shown that ARF17 protein is exclusively detected in the megaspore mother cell, while only weak fluorescence signals from *pARF17::ARF17-GFP* are observed in the chalaza and funiculus of ovules in the *foc* mutant. Additionally, GFP fluorescence is noted in other megaspore mother-like cells [13]. These observations imply that a low level of ARF17 protein in the megaspore mother cell is crucial for its formation, with this expression being suppressed by mature miR160 [13,89]. In situ hybridization studies reveal that mature miR160 is active in various cell types of the ovule, particularly in the megaspore mother cell. Transgenic plants that express a miR160-resistant variant of *ARF17* under its native promoter, *pARF17::mARF17*, generate additional megaspore mother-like cells, which further confirm the necessity of ARF17 in generating a single megaspore mother cell [13].

ARF10 plays a pivotal role in the spatial regulation of germline specification. In *ARF10-GFP* fusion lines, ARF10 expression is limited to the cells surrounding the megaspore mother cell in wild-type plants. However, in miR160-insensitive transgenic lines, ARF10 is distributed throughout the ovule, leading to the formation of supernumerary gametophytes [90]. This indicates that ARF10 regulates the formation of megasporocytes and ensures the generation of a single functional megaspore. ARF10 expression is also coordinated by various genetic networks, including miR160, SEEDSTICK (STK) [91], and ARGONAUTE1 (AGO1) [92], increasing the complexity of the molecular mechanisms underlying germ cell specification [90]. Notably, STK and AGO1 also specifically regulate *SPL/NZZ* expression, suggesting that ARF10 and SPL/NZZ may function within the same regulatory pathway mediated by STK and AGO1.

A localized auxin peak at the apex of the nucellus provides positional information for the formation of the subepidermal megaspore mother cell. The suppression of *ARF17* expression in megasporocytes or their precursors, along with the suppression of *ARF10* expression in the adjacent cells surrounding the megasporocyte, is mediated by miR160. The suppression leads to distinct cell fates: subepidermal cells develop germline identities, while the surrounding cells differentiate into somatic cells. This regulation prevents the formation of additional megasporocytes, maintaining reproductive fidelity [13,89,90,93].

#### 4.2.2. ARFs and SPL/NZZ Interaction in Megasporocyte Formation

SPL/NZZ is essential for the formation of the megasporocyte. In the *spl* mutant, overexpression of *ARF17* inhibits the generation of the megaspore mother cell, suggesting that ARF17 facilitates megasporocyte formation by modulating *SPL/NZZ* expression [13]. Interestingly, the introduction of *pARF17::mARF17* has been shown to rescue the production of the megaspore mother cell, partially. A similar restoration of megasporocyte generation is also noted in *spl foc* double mutants. This indicates that a minimal level of ARF17 activity is necessary for the correct generation of the megasporocyte, and ARF17 regulates the function of SPL/NZZ. On the other hand, the expression of *ARF17* is tightly controlled by miR160, ensuring the regulation of SPL/NZZ function (Figure 2B). Recent studies have also revealed that the ARF10 protein mediates low-level signaling in the cells surrounding the megaspore mother cell, which prevents these cells from differentiating into megaspore mother-like cells [90]. A low level of ARF17 regulates *SPL/NZZ* expression, which then feedback affects the expression of both *PIN1* and *ARF17* [13]. Mature miR160, together with ARF17, confines *PIN1* and *SPL/NZZ* expression, ensuring that the largest subepidermal cell at the apex of the ovule becomes the megaspore mother cell [89].

### 4.3. Auxin-Mediated Regulation of SPL/NZZ in Female Germline Cell Specification

The crosstalk between SPL/NZZ and auxin signaling is important for generating megasporocytes. Auxin provides essential spatial cues through its dynamic distribution within the ovule. It coordinates with SPL/NZZ signaling and ensures the formation of a single megaspore mother cell. This interaction, together with the regulation of ARF transcription factors, guarantees the precise expression of SPL/NZZ and the timely specification of the female germline cell [13]. Furthermore, the interplay between auxin, SPL/NZZ, and epigenetic regulation fine-tunes this developmental process (Figure 2C) [94].

The formation of megaspore mother cells is significantly influenced by multiple genetic pathways, especially those regulating the expression of *SPL/NZZ*. Retinoblastoma-Related 1 (RBR1), a transcriptional repressor, facilitates megasporocyte formation through the repression of *WUSCHEL* (*WUS*) gene activity [95]. KIP-Related Proteins (KRPs) and Interactors/Inhibitors of Cyclin-Dependent Kinases (ICKs), known as CDK inhibitors, inhibit the activity of CDKA to prevent the inactivation of RBR1, ensuring proper megasporocyte formation [96]. Dysfunction of KRP/ICK or RBR1 causes mitotic divisions in megasporocytes and their precursor cells, leading to excess megaspore mother cells [95,97]. Additionally, the cytochrome P450 protein KLUH/CYP78A5 (KLU) and a subunit of the SWI2/SNF2-Related 1 (SWR1) chromatin remodeling complex activate the expression of *WRKY28* in surrounding somatic cells, preventing them from adopting megasporocyte identity [98].

The MADS-box transcription factor STK [91], an ovule identity gene, directly activates AGO1 proteins [92] and the RNA-dependent DNA methylation pathway components Domains Rearranged Methyltransferases 1 (DRM1) and DRM2, which suppress the ectopic expression of *SPL/NZZ* at the apex of the nucellus [99]. Furthermore, epigenetic regulation involving RNA-Dependent RNA Polymerase 6 (RDR6) and RNA helicase MNEME (MEM) [100] inhibits *SPL/NZZ* expression through similar mechanisms. These regulations ensure that only one somatic cell specifies into a megaspore mother cell (Figure 2C) [94].

## 5. A Proposed Theoretical Framework for Auxin-Mediated Germline Specification: Future Research Directions and Perspectives

The current understanding of germline specification in *Arabidopsis* underscores the pivotal role of auxin in orchestrating the transition from somatic to germline cells. Key elements in this process include auxin biosynthesis, polar transport, and downstream signaling pathways. Central to this regulatory network is the transcription factor SPL/NZZ, which integrates auxin signals and modulates gene expression necessary for the specification of both male archesporial cells and female megaspore mother cells [3,12,13]. Despite significant advances, several gaps in our understanding remain, particularly regarding the spatiotemporal integration of these signaling cascades and the extent to which these mechanisms are conserved across species.

### 5.1. Integrating Auxin Regulatory Networks: The Proposed Framework

We propose a comprehensive theoretical framework that summarizes the multiple layers of auxin regulation in germline specification. In this framework, auxin homeostasis is regulated by three interrelated modules: auxin synthesis, transport, and response factors, specifically involving TAA1/TAR2, PIN1, and ARFs. These three modules are interconnected through robust feedback loops. For instance, SPL/NZZ not only acts downstream of auxin signaling but also influences auxin distribution by regulating PIN1, TAA1/TAR2, and ARF17 expression. This reciprocal regulation ensures a self-organizing system where auxin distribution is finely tuned to meet the precise developmental demands of germline specification.

#### 5.1.1. Biosynthesis and Local Production

Auxin is synthesized primarily via the TAA1/TAR2-YUCs pathway, generating indole-3-acetic acid (IAA) that serves as the primary signal for germline initiation. Localized auxin production, particularly in the anther and ovule, establishes dynamic distribution critical for spatial patterning. However, the precise contribution of localized biosynthesis versus long-distance transport remains to be fully delineated [24,101].

#### 5.1.2. Polar Transport and Dynamic Distribution

The spatial distribution of auxin, mediated by PIN efflux carriers, establishes maxima that provide positional cues for germ cell specification. In the ovule, the polar localization of PIN proteins ensures that an auxin maximum forms at the apical region of the nucellus, guiding the differentiation of a single megaspore mother cell [13]. In the anther, although the role of PIN-mediated transport is less well characterized, emerging evidence suggests that auxin maximum is also critical for archesporial cell specification [12].

#### 5.1.3. Signal Transduction and Feedback Regulation

Auxin perception via the TIR1/AFB pathway initiates the degradation of Aux/IAA repressors, freeing ARF transcription factors to activate germline-specific genes. Central to this module, SPL/NZZ functions as a master regulator, integrating auxin signals and feeding back to modulate key components of both biosynthesis (e.g., TAA1/TAR2), polar transport (e.g., PIN1), and auxin response factors (e.g., ARF10 and ARF17) [12,13,102]. The TIR1/AFB pathway is clearly involved in the activation of ARF10 and ARF17. However, it remains to be determined whether the recently identified ABP1-TMK complex and other non-canonical pathways provide alternative routes for auxin signal transduction or even contribute additional layers of regulatory control.

### 5.2. Identified Gaps and Future Research Directions

#### 5.2.1. Spatiotemporal Dynamics of Auxin

Although it is established that auxin dynamic distribution is critical for germ cell specification, the precise spatial and temporal dynamics of auxin biosynthesis versus transport remain poorly defined. We will employ advanced imaging techniques, such as live-cell confocal microscopy coupled with auxin-responsive reporters and single-cell transcriptomics, to map the real-time dynamics of auxin in developing anthers and ovules. These approaches will help distinguish the relative contributions of local biosynthesis and long-range transport.

#### 5.2.2. Role of Auxin Signaling

Both canonical and non-canonical auxin signaling pathways, especially those involving the TIR1/AFB complex that liberates key factors such as ARFs to regulate germ cell specification, are implicated in this process. However, their precise roles in germline specification remain unclear. We will utilize genetic and biochemical approaches to dissect the function of ABP1-TMK signaling in germline development. Creating mutants or using RNA interference to selectively disrupt these pathways could reveal their impact on germline fate decisions and help integrate them into the broader framework.

#### 5.2.3. Feedback Mechanisms Involving SPL/NZZ

While SPL/NZZ is recognized as a central integrator of auxin signals, the feedback mechanisms by which SPL/NZZ modulates auxin biosynthesis and transport need further clarification. We will investigate the regulatory feedback loops involving SPL/NZZ using CRISPR/Cas9-mediated gene editing to generate targeted mutations. Chromatin immunoprecipitation (ChIP) coupled with sequencing (ChIP-seq) can identify direct targets of SPL/NZZ, clarifying how it modulates the expression of genes involved in auxin homeostasis.

#### 5.2.4. Conservation Across Species

The current framework is predominantly based on studies in Arabidopsis. It remains unclear whether the same regulatory mechanisms govern germline specification in other species. We will extend comparative studies to major crops such as rice, maize, and wheat. Using cross-species genetic and molecular analyses, including comparative transcriptomics and functional validation in crop species, will determine the conservation of these mechanisms. Such studies could have profound implications for improving fertility and yield in economically important plants. Currently, the mechanism by which SPL/NZZ regulates female germline cell specification has been applied in watermelon breeding to develop seedless varieties [103]. These findings suggest that auxin-based strategies may have even broader potential applications in crop improvement.

#### 5.2.5. Interplay with Other Hormonal Pathways

Germline specification is a multifaceted process likely influenced by crosstalk between auxin and other hormones (e.g., cytokinins, gibberellins). We will investigate the interactions between auxin and other hormonal signaling pathways during germline specification. Integrated multi-omics approaches can unravel how these pathways converge to regulate the transition from somatic to germline cells.

## 6. Conclusions

The precise regulation and dynamic distribution of auxin are fundamental to germline cell specification, which has significant implications for plant fertility and crop improvement. Genetically manipulating key genes such as TAA1, TAR2, YUCs, and SPL/NZZ through techniques like CRISPR-Cas9, it will be possible to enhance crop fertility and even develop seedless varieties, ultimately improving yields and ensuring greater consistency in crop production [103]. Breeding strategies can utilize auxin’s influence to create hybrids with superior traits, including resilience to stress and enhanced nutritional profiles while minimizing male sterility through interactions within pathways like TPD1-EMS1. Furthermore, biotechnological innovations, particularly in synthetic biology, can be employed to engineer auxin-responsive factors that promote sustainable agricultural practices. Additionally, by addressing unresolved questions through advanced imaging techniques and genetic screening, researchers can further optimize auxin dynamics, ensuring that crop development is robust and adaptable. Future research along these lines is essential for bridging fundamental discoveries in *Arabidopsis* with practical applications in agriculture, ultimately contributing to sustainable crop improvement.
